# Xanthones, A Promising Anti-Inflammatory Scaffold: Structure, Activity, and Drug Likeness Analysis

**DOI:** 10.3390/molecules25030598

**Published:** 2020-01-30

**Authors:** Zheling Feng, Xiuqiang Lu, Lishe Gan, Qingwen Zhang, Ligen Lin

**Affiliations:** 1 State Key Laboratory of Quality Research in Chinese Medicine, Institute of Chinese Medical Sciences, University of Macau, Avenida da Universidade, Taipa, Macau 999078, China; yb77508@um.edu.mo (Z.F.); qwzhang@um.edu.mo (Q.Z.); 2 Fuqing Branch of Fujian Normal University, Fuzhou 350300, China; luxiuqiang@iccas.ac.cn; 3 College of Pharmaceutical Sciences, Zhejiang University, 866 Yuhangtang Road, Hangzhou 310058, China; lsgan@zju.edu.cn

**Keywords:** xanthones, anti-inflammation, drug likeness, SwissADME

## Abstract

Inflammation is the body’s self-protective response to multiple stimulus, from external harmful substances to internal danger signals released after trauma or cell dysfunction. Many diseases are considered to be related to inflammation, such as cancer, metabolic disorders, aging, and neurodegenerative diseases. Current therapeutic approaches include mainly non-steroidal anti-inflammatory drugs and glucocorticoids, which are generally of limited effectiveness and severe side-effects. Thus, it is urgent to develop novel effective anti-inflammatory therapeutic agents. Xanthones, a unique scaffold with a 9*H*-Xanthen-9-one core structure, widely exist in natural sources. Till now, over 250 xanthones were isolated and identified in plants from the families Gentianaceae and Hypericaceae. Many xanthones have been disclosed with anti-inflammatory properties on different models, either in vitro or in vivo. Herein, we provide a comprehensive and up-to-date review of xanthones with anti-inflammatory properties, and analyzed their drug likeness, which might be potential therapeutic agents to fight against inflammation-related diseases.

## 1. Introduction

Inflammation is a kind of active defense reaction of organisms to external stimulations, such as infectious microorganisms, or internal processes, such as tissue injury, cell death, and cancer [1,2,3]. However, long-term low-grade inflammation leads to many human diseases, including aging, metabolic disorders, cancer, and neurodegenerative diseases [4,5,6,7]. Thus, the discovery of anti-inflammatory medicines has been and is continuing to be one of the hotspots of pharmaceutical research.

During inflammatory responses, a variety of cytokines and chemokines are released to restore tissue integrity and orchestrate cell infiltration. Tumor necrosis factor-α (TNF-α) is a major pro-inflammatory cytokine that is secreted from various cells and is associated with immune and inflammatory diseases in humans [8]. Interleukin-1β (IL-1β) is another pro-inflammatory cytokine that is crucial for host defense responses to infection and injury [9]. The IL-6 and IL-12 family of cytokines possess both pro- and anti-inflammatory functions [10] while IL-10 is a potent anti-inflammatory cytokine that impedes the action of many pro-inflammatory mediators to maintain tissue homeostasis and attenuate the damage [11]. Alterations in prostaglandin E2 (PGE2) activity are associated with inflammatory diseases. The pathway of PG synthesis starts with the generation of arachidonic acid from cell membrane phospholipids by phospholipase A2 (PLA2). Then, arachidonic acid is converted to PGs by the enzyme cyclooxygenase (COX) [12]. The inducible COX-2 is recognized as the most active mediator during inflammatory processes. Additionally, inducible nitric oxide synthase (iNOS) is highly expressed under inflammatory conditions, which catalyzes the synthesis of nitric oxide (NO) [13]. Because macrophages produce a wide range of biologically active molecules participating in both beneficial and detrimental outcomes in inflammation, therapeutic interventions targeting macrophages and their products have attracted lots of attention for controlling inflammatory diseases.

**Figure 1:**
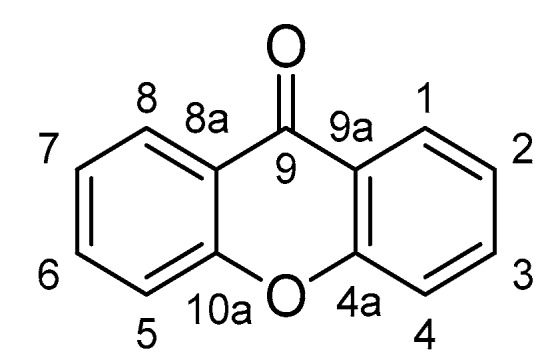
The core structure of xanthone.

Currently, anti-inflammatory therapy mainly includes non-steroidal anti-inflammatory drugs (NSAIDS) and glucocorticoids, both of which possess various side effects, such as cardiotoxicity, hepatotoxicity, and immunological dysfunction [14,15]. Natural products have attracted increasingly more attention due to their safety and effectiveness [16]. Emerging evidence indicates that natural products always function as multi-component and multi-target patterns [17]. Naturally occurring anti-inflammatory compounds might be promising candidates for the treatment of enteritis, arthritis, and skin inflammation. Xanthones were firstly isolated in 1855 by a German scientist pursuing research on dysentery and then named by the Greek word for yellow, xanthos [18]. Xanthones possess a unique 9*H*-Xanthen-9-one scaffold (Figure 1), which mainly occurs in the plants of the families Gentianaceae and Hypericaceae, as well as some fungi and lichens [19]. Several types of xanthones have been identified, including simple oxygenated xanthones, xanthone glycosides, prenylated xanthones, xanthonolignoids, and miscellaneous [20]. The studies of xanthone are provoking not only due to the structural diversity but also a variety of pharmacological activities. Many xanthones have been reported with potent anti-inflammatory properties [21,22,23,24,25]. Herein, we provided a comprehensive and up-to-date review of xanthones with anti-inflammatory properties and analyzed their drug likeness, which might be further developed to treat inflammation-related diseases.

## 2. Xanthones with Anti-Inflammatory Properties

**Figure 2:**
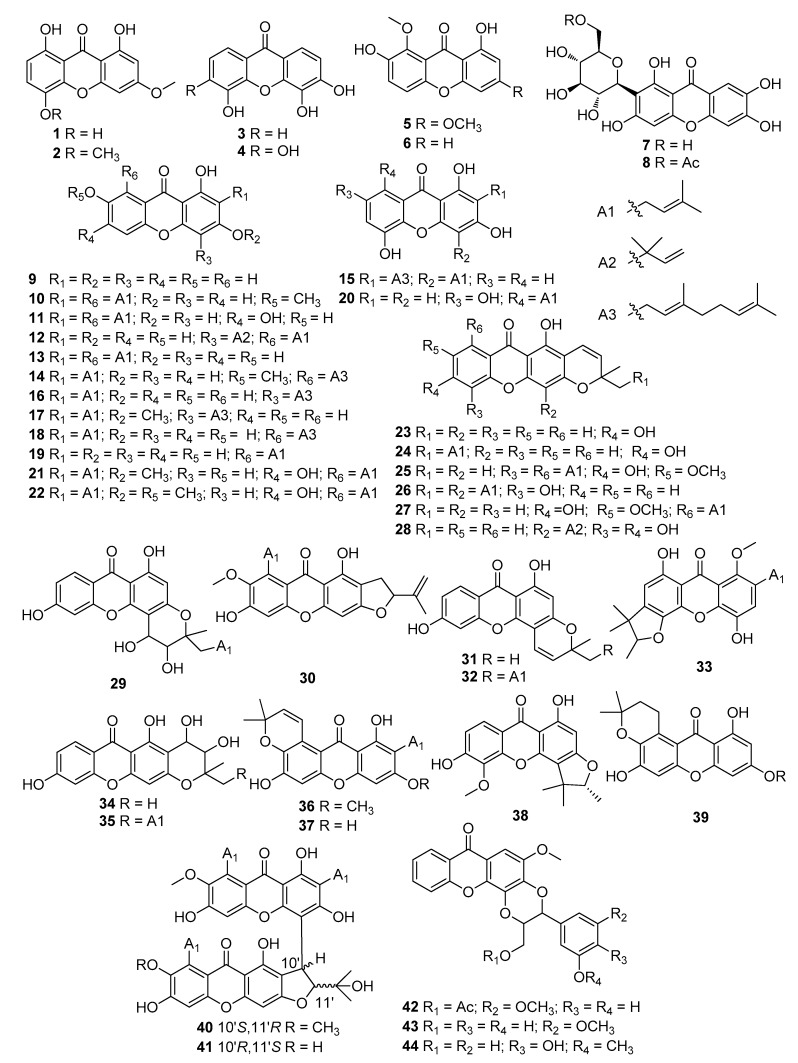
Structures of xanthones with anti-inflammatory activity. The symbol Ac represents an acetyl group.

Using the keywords xanthone and inflammation, we collected data from Google Scholar, Web of Science, Scopus, and Pubmed. A total of 44 xanthones were found with anti-inflammatory properties, containing 6 simple oxygenated xanthones (**1**–**6**), 2 xanthone glycosides (**7**, **8**), 33 prenylated xanthones **(9**–**41**), and 3 xanthonolignoids (**42**–**44**) (Figure 2). Many models, either in vitro or in vivo, have been recruited to evaluate the anti-inflammatory properties of xanthones. To organize the review, the xanthones were classified based on bioassays (Table 1).

Macrophages are a major component of the mononuclear phagocyte system [26]. Monocytes migrate into various tissues and transform into macrophages. Macrophages play a critical role in the initiation, maintenance, and resolution of inflammation. Lipopolysaccharide (LPS), a component of the Gram-negative bacterial cell wall, has been widely used to induce an inflammatory response in macrophages [27]. The LPS-stimulated RAW264.7 macrophage model is an effective tool for anti-inflammatory drug screening and anti-inflammatory mechanism investigation. Using a LPS-induced RAW264.7 macrophage model, 3,4,5,6-tetrahydroxyxanthone (**4**) and mangiferin (**7**) were found to suppress the generation of TNF-α and intercellular adhesion molecule-1 (ICAM-1) [28]. Six xanthones, including 1,3,6,7-tetrahydroxy-8-prenylxanthone (**19**) [29], β-mangostin (**22**) [30], nagostenone F (**30**) [31], inophinnin (**33**) [32], garcinoxanghone B (**40**) [33], and garcinoxanthone C (**41**) [33], were reported to reduce NO production in LPS-stimulated RAW264.7 macrophage. 6′-*O*-acetyl mangiferin (OAM) (**8**), an acetylated xanthone C-glucoside, was reported to suppress iNOS and COX-2 expression, thereby inhibiting the levels of TNF-α, IL-1β, and IL-6 in LPS-stimulated RAW264.7 cells. Furthermore, OAM inhibited the LPS-induced phosphorylation of c-Jun N-terminal kinases (JNK), extracellular signal-regulated kinase (ERK), and p38, which led to the blockade of nuclear factor-κB (NF-κB) and inhibitor κB (IκB)-α activation [34]. Cudratricusxanthone A (**12**), isolated from the roots of *Cudrania tricuspidata* Bureau (Moraceae), was found to induce heme oxygenase-1 (HO-1) expression at a non-cytotoxic concentration (1–10 μmol/L) in LPS-treated RAW264.7 macrophages, which in turn suppressed PGE2, NO, TNF-𝛼, and IL-1β production [35]. Dual arachidonate 5-lipoxygenase (5-LOX) and COX inhibitors are potential new drugs to treat inflammation. 6-Dihydroxy-5-methoxy-4′,5′-dihydro-4′,4′,5′-trimethylfurano-(2′,3′:3,4)-xanthone (**38**) inhibited COX-1 and COX-2 production, and 5-LOX mediated leukotriene B4 (LTB4) formation in LPS-induced RAW264.7 macrophages [36]. Among the xanthones isolated from the roots of *Cratoxylum formosum* ssp. *pruniflorum*, several compounds (**6**, 1**5**–**18**, **21**, **26**–**28**, and **37**) showed anti-inflammatory activity in LPS-induced RAW 264.7 macrophages [37]. Dulxisxanthone F (**21**) was found to downregulate the mRNA expression of iNOS and COX-2 in dose-dependent manners, and 5,9-dihydroxy-8-methoxy-2,2-dimethyl-7-(3-methylbut-2-enyl)-2*H*,6*H*-pyrano-[3,2*b*]-xanthone (**27**) only inhibited the mRNA expression of iNOS but not COX-2. Two xanthones, ellidifolin (**1**) and swerchirin (**2**), were isolated from *Swertia chiraytia* [38], which were found to inhibit the production of pro-inflammatory cytokines IL-6 and TNF-𝛼 in LPS-stimulated RAW264.7 macrophages; furthermore, ellidifolin (**1**) inhibited the production of PGE2 by suppressing the phosphorylation of JNK, ERK, and p38 MAPKs (mitogen-activated protein kinases).

Interferon γ (IFN𝛾) is a dimerized soluble cytokine, and aberrant IFN𝛾 expression is related to a number of inflammatory and autoimmune diseases [39]. LPS plus IFN𝛾 stimulation caused the increase of TNF receptor associated factor family member-associated NF-κB activator binding kinase 1 (TBK1) expression, p50/p65 nuclear translocation, and activation of NF-κB in RAW264.7 macrophages, 1,3,5,7-tetrahydroxy-8-prenylxanthone (**20**), reversed the above changes to suppress the production of IL-6, IL-12, and TNF-𝛼 [40]. In IFN𝛾 plus LPS-induced RAW264.7 macrophages, hyperxanthone E (**39**) was reported to decrease NO production [40].

The major role of neutrophils in the host defense is to eliminate invading microorganisms [41]. In neutrophils, *N*-formylmethionyl-leucyl-phenylalanine (fMLP) is a powerful activator of polymorphonuclear and mononuclear phagocytes, and the effects of fMLP on neutrophil activity can be inhibited by pertussis toxin [42]. The neutrophil-mediated inflammatory response is regarded as a multi-step process involving the initial adhesion of circulating neutrophils to activated vascular endothelium [43]. In fMLP/CB-stimulated human neutrophils, several gambogic acid analogs (**23**, **24**, **29**, **31**, **32**, **34**, and **35**) inhibited superoxide anion generation and elastase release [44]. Several xanthons (**3**, **9**, **42**, and **43**) were isolated from the twigs of *Hypericum oblongifolium* wall, which showed anti-inflammatory activity in isolated human neutrophils [45].

CD3^−^ synovial cells are suggested to play an important role in RA development and therefore are a perfect model in the search for new anti-arthritic drugs. Mangiferin (**7**) downregulated TNF-α, IL-1β, and IFN-γ expression in TNF-α-stimulated CD3^−^ synovial cells from rheumatoid arthritis (RA) patients, which indicated that mangiferin could be a potent candidate for the treatment of RA [46].

Sepsis is a major cause of death worldwide [47]. Infection-induced inflammation is strongly regulated by many endogenous negative feedback mechanisms that modulate the intensity of inflammation, promote its eventual resolution, and return it back to homeostasis. Mangiferin (**7**) dose-dependently upregulated the expression and activity of HO-1 in the lung from septic mice [48].

Carrageenan is a pro-inflammatory agent used as a tool to induce inflammatory hyperalgesia in rats and mice [49]. The carrageenan-induced peripheral inflammatory pain model is widely used because it resembles inflammatory pain susceptible to both steroidal and nonsteroidal anti-inflammatory drugs [50]. Local administration of mangiferin (**7**) prevented inflammatory mechanical hyperalgesia induced by carrageenan in rats, which depended on the inhibition of TNF-α production/release and the CINC1 (cytokine-induced neutrophil chemoattractant 1)/epinephrine/PKA (protein kinase A) pathway [51].

MC3T3 is an osteoblast precursor cell line derived from *Mus musculus* (mouse), which is one of the most convenient and physiologically relevant systems for the study of transcriptional control in calvarial osteoblasts [52]. Dexamethasone is a known synthetic glucocorticoid, which induces sodium-dependent vitamin C transporter in MC3T3-E1 cells [53]. Bone morphogenetic protein 2 (BMP2) plays a role in postnatal bone formation, mediated by activating ligand-bound Small Mothers Against Decapentaplegic (SMAD) family members [38]. Mangiferin (**7**) attenuated dexamethasone-induced injury and inflammation in MC3T3-E1 cells by activating the BMP2/Smad-1 signaling pathway [54].

HFLS-RA is a human fibroblast-like synoviocyte with high proliferating ability and susceptibility. HFLS-RA cell is an excellent cellular model for studying synoviocyte physiology in relation to the development and treatment of RA [55]. α-Mangostin (**10**) (10 μg/mL) was found to suppress the expression and activation of key proteins in the NF-κB pathway and inhibit the nuclear translocation of p65 in HFLS-RA cells [56].

Adjuvant-induced arthritis (AA) is evaluated by paw edema, arthritis score, and hematological parameters. α-Mangostin (**10**) protected joints from rats suffering from AA, indicated by attenuated paw swelling, reduced inflammatory cell infiltration, decreased secretion of IL-1β and TNF-α in serum, and inhibition of NF-κB activation in synovia [56]. 

The presence of neuroinflammation is a common feature of dementia [57]. Reactive microgliosis, oxidative damage, and mitochondrial dysfunction are associated with the pathogenesis of all types of neurodegenerative dementia, such as Parkinson’s disease dementia (PDD), frontotemporal dementia (FTD), Alzheimer’s disease (AD), and Lewy body dementia (LBD). Peripheral LPS-induced neuroinflammation in C57bl/6J mice has been used to evaluate neuroinflammation and neurodegeneration as an adjuvant therapeutic strategy. α-Mangostin (**10**) reduced the levels of proinflammatory cytokine IL-6, COX-2, and 18 kDa translocator protein (TSPO) in the brain from LPS-induced neuroinflammation in C57BL/6J mice, which was considered as an adjuvant treatment in preclinical models of AD, PD, and multiple sclerosis [58].

RA is a long-term autoimmune disease in which the body’s immune system mistakenly attacks the joints; RA causes pain, stiffness, and swelling in the joints [59]. α-Mangostin (**10**) decreased the clinical score at both doses (10 and 40 mg/kg) and decreased the histopathological score at the high dose in collagen-induced arthritis (CIA) in DBA/1J mice [60].

Asthma is a chronic inflammatory disease of the airways characterized by reversible airway obstruction, airway hyperreactivity (AHR), and remodeling of the airways [61]. Allergic asthma is associated with excessive T helper type 2 (Th 2) cell activation and AHR [55]. α-Mangostin (**10**) and γ-mangostin (**9**) reduced the major pathophysiological features of allergic asthma in ovalbumin-induced allergic asthma mice, including inflammatory cell recruitment into the airway, AHR, and increased levels of Th2 cytokines and phosphoinositide 3-kinase (PI3K) activity, which indicated both compounds might have therapeutic potential for the treatment of allergic asthma [62].

3T3-L1 cells are fibroblasts derived from mice, which have an extraordinary tendency to accumulate lipids. 3T3-L1 cells have an adipocyte morphology, acquire the signet ring appearance of adipose cells, and are sensitive to lipogenic and lipolytic hormones, which are used in many types of research on adipose tissue [63]. α-Mangostin (**10**) inhibited NF-κB, and NFR2 in 3T3-L1 preadipocytes [64]. γ-Mangostin (**7**), a minor xanthone from mangosteen, inhibited NF-κB transcription activity and secretion of monocyte chemotactic protein-1 (MCP-1) in 3T3-L1 adipocytes [54]. 1,3,6,7-Tetrahydroxy-8-prenylxanthone (**19**) was reported to ameliorate TNF-α-mediated inflammation in 3T3-L1 adipocytes, through inhibiting MAPKs and NF-κB activation and promoting sirtuin 3 expression [29]. 

In acetic acid-induced mice, 2,8-dihydroxy-1,6-dimethoxyxanthone (**5**), isolated from the leaves and twigs of *Eunoymus alatus*, reduced paw edema [65]. 

Ethyl phenylpropionate (EPP) is a compound used to induce ear edema [66]. Several xanthones were isolated from the acetone extract of the twigs of *Garcinia cowa* (Guttiferae), and α-mangostin (**10**), cowaxanthone B (**13**), cowaxanthone C (**25**), cowaxanthone D (**14**), and cowanin (**13**) exhibited anti-inflammatory activity in an ear edema mouse model [67].

Adipose tissue inflammation is a dynamic process controlled by multiple mechanisms [68]. In LPS-induced adipose tissue inflammation mice, α-Mangostin (**10**) alleviated adipose tissue inflammation by reducing the macrophage content and shifting the pro-inflammatory macrophage polarization [18]. 1,3,6,7-tetrahydroxy-8-prenylxanthone (**19**) inhibited LPS-mediated inflammation through inhibiting MAPKs and NF-κB activation and promoting sirtuin 3 expression [29].

## 3. Comparison of the Drug Likeness of Anti-Inflammatory Xanthones with Marketed Drugs

Swiss Institute of Bioinformatics provides SwissADME to calculate molecular descriptors of the identified anti-inflammatory xanthones [70]. For each compound, the following descriptors were calculated: Molecular weight (MW); number of stereogenic centers; number of hydrogen bond acceptors (HBA) and donors (HBD), described as the electrostatic bond between a hydrogen and a lone pair of electrons; number of rotatable bonds (RB); number of rings; fraction of sp^3^ carbons (Fsp^3^) defined as the ratio of sp^3^ hybridized carbons over the total number of carbons; and fraction of aromatic heavy atoms (Far), defined as the number of aromatic heavy atoms divided by the total number of heavy atoms [68].

**Figure 3:**
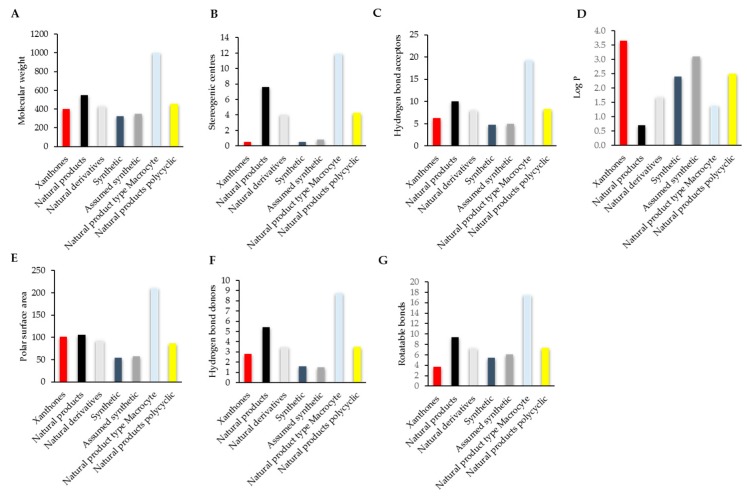
Mean values of MW (**A**), stereogenic centers (**B**), Log P (**C**), HBA (**D**), HBD (**E**), PSA (**F**), and rotatable bond (**G**) for anti-inflammatory xanthone derivatives (red), natural products (black), natural derivatives (light grey), synthetic (dark blue), assumed synthetic (dark grey), natural product type macrocyte (light blue), and natural products polycyclic (yellow).

The obtained values for each molecular descriptor are shown in Table S1 (Supplementary Materials), grouped according to the categories defined in the previous section. Drug development involves the assessment of absorption, distribution, metabolism, and excretion (ADME), drug-likeness, and medicinal chemistry friendliness. Physicochemical properties, pharmacokinetics, polar surface area (PSA), Log S and iLOGP, and bioavailability properties for xanthone derivatives are presented in Table S2 (Supplementary Materials). Especially for log P and log S, more than one algorithm was used in the process. Seven molecular descriptors were calculated, including the mean and median values for anti-inflammatory xanthone derivatives (Figure 3). 

For the sake of comparison between the chemical properties of the anti-inflammatory xanthone derivatives and marketed drugs, these compounds were divided into synthetic compounds, assumed synthetic compounds, natural product-type macrocycles, polycyclic compounds, natural products, and natural product derivatives [71] (Figure 3).

### 3.1. Size: Molecular Weight

Traditional therapeutic agents are small molecules that fall within the Lipinski’s rule of five [72], including a molecular mass less than 500 Da, no more than 5 HBD, no more than 10 HBA, and an octanol-water partition coefficient logP not great than 5. According to the results, the mean molecular weight for anti-inflammatory xanthone derivatives was 401.3 Da, which adhered to Lipinski’s rule (Figure 3B). Most NSAIDS typically adjust to Lipinski’s rule, with a molecular mass of less than 500 Da [73]. Among all the reviewed anti-inflammatory xanthone derivatives, about 95% of them have a molecular weight less than 500 Da, except two dimers.

### 3.2. Chirality: Number of Stereogenic Centers

Because the core structure of xanthone is planar, the number of stereogenic centers in xanthones was less than that of synthetic compounds, assumed synthetic compounds, natural product-type macrocycles, polycyclic compounds, natural products, and natural product derivatives [71]. The average number of the stereogenic center is 0.5 for the identified anti-inflammatory xanthone derivatives (Figure 3B). The highest value of the stereogenic center is natural product-type macrocycles, with a mean value of 12.0. For the synthesis of new drugs, the more chiral centers, the more difficult and costly the synthesis is. The mean value of the identified anti-inflammatory xanthone derivatives is satisfied with the new drug development criteria.

### 3.3. Polarity: PSA and HBD/HBA

Prediction of the permeability is a major challenge in drug discovery. Solubility governs the skill of drugs to transport across systemic circulation, brain penetration, and the gastrointestinal membrane. Polarity is highly relevant to the prediction of permeability, and PSA is used in the practice of medicinal chemistry to quantify polarity [74]. PSA is defined as the surface area of a molecule that arises from oxygen or nitrogen atoms, plus hydrogen atoms attached to nitrogen or oxygen atoms. The PSA principle takes into account the contribution to polarity, arising from electronegative atoms different from nitrogen and oxygen, but as different atoms have different electronegativity, they will produce a redistribution of the electron density. Thus, some drugs are neglected in PSA calculation. PSA does not distinguish HBD from HBA properties and shows a high degree of correlation with the number of HBA groups but lower correlation with the number of HBD groups [75]. PSA is widely used with discrete success as a molecular descriptor model of permeability and other ADME-related properties to obtain a better understanding and thus prediction of biological events influenced by polarity.

**Figure 4:**
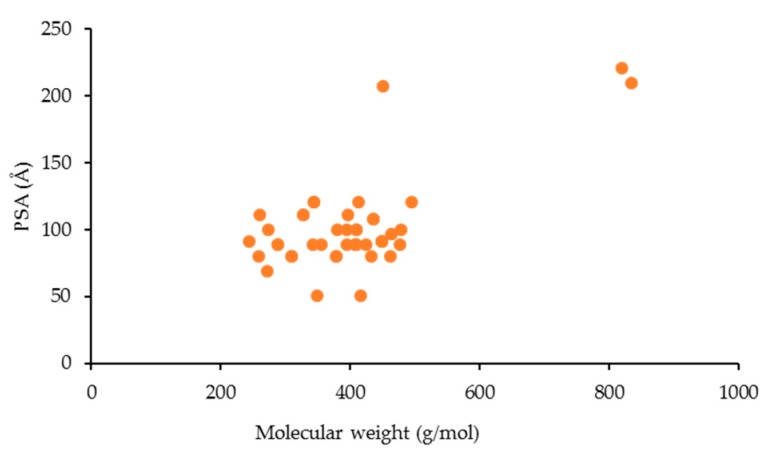
PSA values of the anti-inflammatory xanthone derivatives vs. the molecular weight (MW).

The PSA mean values were 99.4 Å^2^ for xanthone derivatives, 86.9 Å^2^ for polycyclic new drugs, and 105.3 Å^2^ for natural products. Similarly, the HBA/HBD and PSA values for the anti-inflammatory xanthone derivatives increased accompanied by an increase of the molecular weight (Figure 3D–F and Figure 4). According to the rules of five (Ro 5), HBD < 5, HBA < 10, and PSA < 140 Å^2^ [76], most of the anti-inflammatory xanthone derivatives satisfied this criterion, which indicated that they might have good oral absorption.

### 3.4. Molecular Flexibility: Rotatable Bonds and Aromatic Character

RBs are defined as any single bond, not in a ring, bound to a nonterminal heavy atom. The amide C-N bonds are excluded because of their high rotational energy barrier. Reduced molecular flexibility, as measured by the number of RBs, and low PSA or total HB are important predictors of good oral bioavailability [77]. The RB number was found to influence oral bioavailability, with 65% of compounds with ≦7 RBs exhibiting an oral bioavailability of ≧20% [78]. The increased RB number has a negative effect on the permeation rate. A threshold permeation rate is a prerequisite of oral bioavailability.

The mean number of RBs for the anti-inflammatory xanthone derivatives is 3.7, and the mean number of aromatic heavy atoms is 14.8. The mean values of RBs for the polycyclic compounds, natural products, natural product derivatives, and synthetic drugs are 7.4, 9.4, 7.4, and 5.4, respectively. The RBs for most of the identified anti-inflammatory xanthone derivatives are less than those of polycyclic natural products, indicating a good permeation rate (Figure 3G). Compared to synthetic compounds (mean Fsp^3^ of 0.27), natural products (mean Fsp^3^ of 0.55) are more like a typical trait [70]. The identified anti-inflammatory xanthone derivatives have a mean Fsp^3^ of 0.24 because xanthone derivatives have a higher aromatic character. 

### 3.5. Lipophilicity: LogP

The major role of lipophilicity in drug discovery is to balance potency and ADME properties [79]. Lipophilicity is commonly described as logD, where the distribution coefficient, D, is quantified by the concentration of all species (unionized and ionized) of a compound at a given pH in two immiscible phases (commonly 1-octanol and water/buffer) at equilibrium. The distribution coefficient (D) is replaced with the partition coefficient (P) at any given pH if only one species (typically neutral) is present.

**Figure 5:**
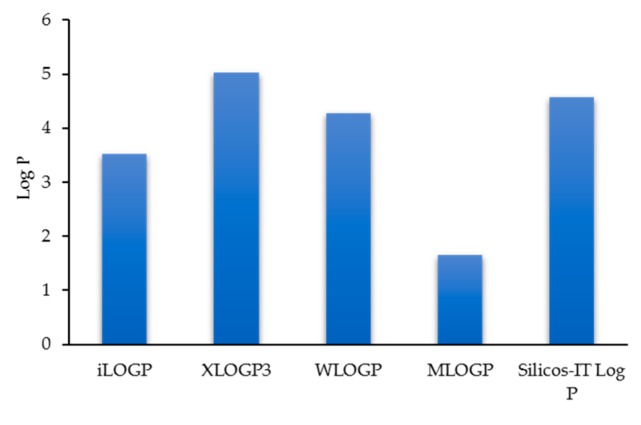
Mean bars log P values of each category of anti-inflammatory xanthone derivatives calculated by different methods.

The log P values of the anti-inflammatory xanthone derivatives vary a lot depending on the predict method on Swiss ADME. MLOGP is the most discrepant in all the logP index (Figure 5). Compared to the natural products, natural derivatives, synthetic compounds, assumed synthetic compounds, natural product-type macrocycles, and natural product polycyclic, the logP value of the anti-inflammatory xanthone derivatives (3.7) is higher, which indicated a lower oral bioavailability.

### 3.6. Solubility: Log S

It has been reported that over 75% of drug candidates have low solubility based on the biopharmaceutics classification system (BCS). Solubility is one of the challenging properties in drug discovery. Compounds that are not fully soluble in bioassays result in erratic assay results, such as enzyme and cell-based assay. Because the actual concentration in solution is much lower than the target concentration, it can appear as an artificially low potency. Solubility issues cause a lot of frustration and lots of productivity in drug discovery [80]. In some cases, a high amount of organic solvent has to be used to dissolve the compounds, which causes an unexpected toxicity. The development of insoluble compounds can be expensive and time consuming. Solubility is expressed as log S and values greater than –4 are acceptable for a drug [81].

**Figure 6:**
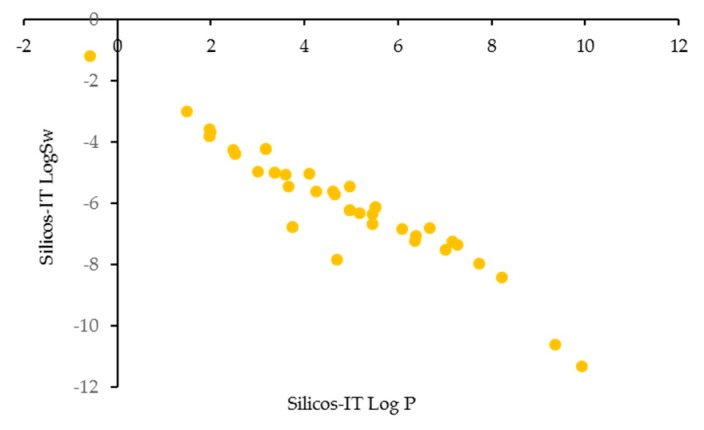
Log S (SILICOS-IT) of the anti-inflammatory xanthone derivatives vs. LogP (SILICOS-IT).

The relationship between the molecular size and aqueous solubility of xanthone derivatives is fairly stable; when the molecular weight gets higher, the solubility of anti-inflammatory xanthone derivatives decreased (Figure 6). Most anti-inflammatory xanthone derivatives might face the solubility issue.

## 4. Compliance of Xanthones with the Rules of Drug Likeness

In order to quickly eliminate lead candidates that have poor physicochemical properties for oral bioavailability, the five rules of drug likeness have been widely adopted in the pharmaceutical industry, which helps to predict the in vivo behavior of potential drugs [77]. The biophysicochemical properties and molecular descriptors of the anti-inflammatory xanthone derivatives were framed as different rules of compliance. Most anti-inflammation xanthone derivatives appear to have a good drug likeness, which are green in the visualization map in Table S3 (Supplementary Materials).

## 5. Trends on the PK Behavior of Xanthones

The brain or intestinal estimated permeation method (BOILED-Egg) is proposed as an accurate predictive model that works by computing the lipophilicity and polarity of small molecules [82]. It delivers a rapid, intuitive, and easily reproducible yet statistically unprecedented robust method to predict the passive gastrointestinal (GI) absorption and brain access of small molecules useful for drug discovery and development [83].

**Figure 7:**
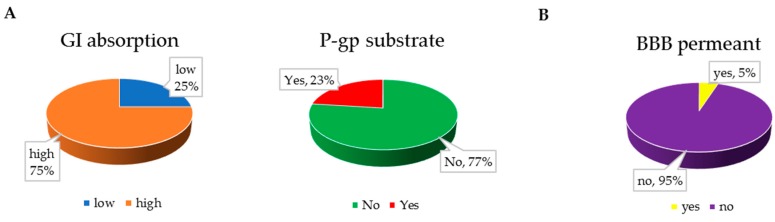
(**A**) GI absorption for the identified anti-inflammatory xanthone derivatives (left pie chart). Anti-inflammatory xanthone derivatives with high GI absorption were classified accordingly to its P-gp substrate (right pie chart). (**B**) BBB permeability of the identified xanthone derivatives.

According to the results, about 75% of anti-inflammatory xanthone derivatives have a higher probability of being highly absorbed in the GI (Figure 7A). It might be due to their lower MW and lower polarity of the benzene rings. In total, 33 anti-inflammation xanthone derivatives have higher GI absorption, and 10 xanthone derivatives have a high probability of being a substrate for P-glycoprotein (P-gp, Figure 7A). 

The blood–brain barrier (BBB) is a highly selective semipermeable border that separates the circulating blood from the brain and extracellular fluid in the central nervous system [84]. Most of the anti-inflammatory xanthone derivatives have a low probability of being able to cross the BBB (Figure 7B), and there are 10 xanthone derivative with potential abilities to be a substrate for P-gp (Table S5, Supplementary Materials).

**Figure 8:**
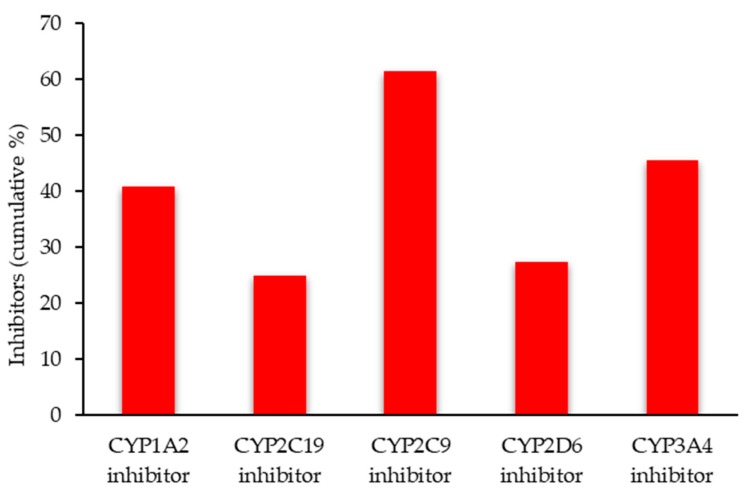
CYP450 enzyme inhibitors of the anti-inflammatory xanthone derivatives.

SwissADME provides the potential ability of xanthone derivatives to be a P-gp substrate to inhibit one of five major isoforms of cytochrome P450, CYP450 (CYP1A2, CYP2C19, CYP2C9, CYP2D6, and CYP3A4) [85,86]. The predicted results are shown in Table S4 (Supplementary Materials). The anti-inflammatory xanthone derivatives have higher opportunities to be CYP450 enzyme inhibitors, especially for the CYP2C9 (Figure 8). Compound **35** was identified as a possible inhibitor of all the CYP isoforms (Table S4, Supplementary Materials).

## 6. Conclusions

Xanthones have been implicated in biological activities and chemical isolation, as well as total synthesis. In the last decade, increased reports of xanthones as potential anti-inflammatory reagents have been challenged in the phytochemical, pharmacological, and synthetic community to innate challenges of the construction of this class of natural products. However, although most of the recent research has concentrated on anti-inflammatory activities in vitro and their mechanisms, in vivo information is still restricted and lacks good-quality preclinical models to make a further step in clinical application. More efforts should be paid to verify the therapeutic effects of xanthones using in vivo animal models. Besides mangiferin and α-mangostin, there is a hint of the emergence of studies from other xanthones concerning the discovery of drug candidates.

So far, there are still limited data available on the bioavailability of xanthones. The lack of toxicity studies on xanthones does not negate its importance, as the safety and efficacy of drugs are related to each other. Future structure–activity relationship studies on simplified fragments of the members of this natural product family are also necessary to ascertain both the key features related to activity and the mode of action of these natural products. Ongoing exciting results remain to be discovered and reviewed. Future research on the chemistry and biology on anti-inflammatory xanthones looks very bright and challenging, and with tremendous therapeutic applications.

By using the online bioinformatics tool SwissADME, the biophysicochemical properties, molecular descriptors, and PK parameters were predicted and evaluated for xanthones with anti-inflammatory properties. A series of drug-likeness analysis methods and parameters were mentioned to proceed with the anti-inflammatory xanthone derivatives, such as logP, MW, logS, HBA, HBD, PSA, number of stereogenic centers, and RBs, even with CYP450 inhibitors. Xanthone derivatives have good compliance with the drug-likeness chemical properties. Many new drugs were developed from natural products and natural plants. Experimental data combined with bioinformatics predictive tools could be an efficient and economical way to discover new health products and new anti-inflammatory drugs. Despite some compounds not obeying the usual drug-likeness rules, many others have been successfully developed as new drugs.

## Figures and Tables

**Table 1:** Xanthones with anti-inflammatory activity.

Model/Method	No	Dose	Outcomes	Ref.
LPS-stimulated RAW264.7 macrophages	**1**	10 μmol/L	Suppressed the phosphorylation of IKK-β, Akt, and p65	[38]
	**2**	10 μmol/L	Inhibited the production of IL-6 and TNF-α	[38]
	**4, 7**	25, 50 μg/mL	Suppressed the generation of TNF-α and ICAM-1	[28]
	**6, 15**−**18, 21, 26**−**28, 37**	3, 10, 30, 100 μmol/L	Downregulated mRNA expressions of iNOS and COX-2	[37]
	**9**	50 μmol/L	Suppressed iNOS, COX-2, inhibited TNF-α, IL-1β, IL-6, IκB-α	[34]
	**12**	1, 2, 5, 10 μmol/L	Induced HO-1 expression and increased HO-1 activity, inhibited TNF-α, IL-1β	[35]
	**19**	5, 10, 20 μmol/L	Inhibited NO production and IL-6 secretion	[29]
	**22**	11.72 ± 1.16 μmol/L	Inhibited NO production	[30]
	**30**	20, 40, 60 μmol/L	Inhibited the production of NO, iNOS, TNF-α, IL-6, and IL-1β	[31]
	**33**	6.25 μmol/L	Suppressed NO production	[32]
	**38**	50 μg/mL	Inhibited COX-1, COX-2 and 5-LOX-mediated LTB4 formation	[36]
	**40**	11.3 ± 1.7 μmol/L	Inhibited NO production	[33]
	**41**	18.0 ± 1.8 μmol/L	Inhibited NO production	[33]
LPS/IFN𝛾-stimulated RAW264.7 macrophages	**20**	3.125–25 𝜇mol/L	Suppressed IL-6, IL-12, and TNF-𝛼	[69]
	**39**	10 μmol/L	Decreased NO production	[40]
Human neutrophils	**3, 7, 42, 43**	1000 μg/mL	Inhibited WST-1 by NADPH oxidase	[45]
	**23, 24, 29, 31, 32, 34, 35**	10 μg/mL	Inhibited superoxide anion generation and elastase release	[44]
CD3^−^ synovial cells	**7**	100 μg/mL	Downregulation of TNF-α, IL-1β, and IFN-γ	[46]
Lung of septic mice		10, 30, 100 mg/kg	Upregulated the expression and activity of HO-1	[48]
Carrageenan-induced mechanical hyperalgesia Wistar rats		100 μg/paw	Inhibited TNF-α level through CINC-1/epinephrine/PKA pathway	[51]
MC 3T3-E1 cell line		10, 20, 30, 40 μmol/L	Alleviated oxidative stress by activating the BMP2/Smad-1 signaling pathway	[54]
HFLS-RA cells	**10**	10 μg/mL	Inhibited nuclear translocation of p65	[56]
AA rats	**10**	2.5−10 μg/mL	Inhibited fibrous hyperplasia, synovial angiogenesis, cartilage	[56]
Peripheral LPS-induced neuroinflammation in C57BL/6J mice	**10**	40 mg/kg	Reduced brain levels of IL-6 and COX-2	[58]
Established CIA in DBA/1J mice	**10**	10, 40 mg/kg	Reduced the levels of anti-collagen IgG2a and autoantibodies in serum and the production of LIX/CXCL5, IP-10/CXCL10, MIG/CXCL9, RANTES/CCL5, IL-6 and IL-33 in joints	[60]
Ovalbumin-induced allergic asthma mice	**9, 10**	10, 30 mg/kg	Increased Th2 cytokine	[62]
3T3-L1 cells	**10, 19**	10 μmol/L	Inhibited PPARγ and NFR2 through NF-κB	[64]
Acetic acid-induced mice	**5**	10, 20 mg/kg	Reduced paw edema	[65]
EPP-induced ear edema	**10, 13, 14, 25, 36**	1 mg/kg	Inhibited edema	[67]
LPS-induced adipose tissue inflammation mice	**10**	10 mg/kg	Reduced macrophage content and shifted pro-inflammatory macrophage polarization	[18]
	**19**	20 mg/kg	Reduced macrophage content through inhibiting MAPKs and NF-κB activation	[29]

## Supplementary Materials

The following are available online, Table S1: Molecular descriptors of anti-inflammatory xanthones. Table S2: Biophysiochemical properties of anti-inflammatory xanthones. Table S3: Violations of drug-likeness rules by the anti-inflammatory xanthones. For each compound, the type of violations of each rule was described.
