# Supplementary material for: Xanthones, A Promising Anti-Inflammatory Scaffold: Structure, Activity, and Drug Likeness Analysis
Source: Molecules. 2020 Jan 30;25(3):598. doi: 10.3390/molecules25030598 (PMC7037265; doi:10.3390/molecules25030598)
Supplement: Supplementary file 1 [file molecules-25-00598-s001.pdf]

Table S1. Molecular descriptors of anti-inflammatory xanthones.

No	Formula	MW	Stereogenic Centers	#H-bond acceptors	#H-bond donors	#Rotatable bonds	rings	Fraction Csp3	FAr
1	$C_{14}H_{10}O_6$	274.23	0	6	3	1	3	0.07	0.70
2	$C_{15}H_{12}O_6$	288.25	0	6	2	2	3	0.13	0.67
3	$C_{15}H_{12}O_5$	272.25	0	5	1	2	3	0.13	0.70
4	$C_{13}H_8O_6$	260.2	0	6	4	0	3	0	0.74
5	$C_{15}H_{12}O_6$	288.25	0	6	2	2	3	0.13	0.67
6	$C_{14}H_{10}O_5$	258.23	0	5	2	1	3	0.07	0.74
7	$C_{23}H_{24}O_7$	412.43	3	7	4	3	4	0.35	0.47
8	$C_{19}H_{18}O_{12}$	438.34	0	12	8	3	5	0.32	0.45
9	$C_{13}H_8O_5$	244.2	0	5	3	0	3	0	0.78
10	$C_{24}H_{26}O_6$	410.46	0	6	3	5	3	0.29	0.47
11	$C_{20}H_{18}O_{12}$	450.35	0	12	7	3	5	0.3	0.44
12	$C_{23}H_{24}O_6$	396.43	0	6	4	4	3	0.26	0.48
13	$C_{20}H_{20}O_6$	356.37	0	6	2	4	3	0.25	0.54
14	$C_{24}H_{24}O_6$	408.44	0	6	2	3	4	0.29	0.47
15	$C_{28}H_{32}O_5$	448.55	0	5	3	7	3	0.32	0.42
16	$C_{28}H_{32}O_5$	448.55	0	5	3	7	3	0.32	0.42
17	$C_{29}H_{34}O_5$	462.58	0	5	2	8	3	0.34	0.41
18	$C_{28}H_{32}O_5$	448.55	0	5	3	7	3	0.32	0.42
19	$C_{18}H_{16}O_6$	328.32	0	6	4	2	3	0.17	0.58
20	$C_{18}H_{16}O_6$	328.32	0	6	4	2	3	0.17	0.58
21	$C_{24}H_{26}O_6$	410.46	0	6	3	5	3	0.29	0.47
22	$C_{25}H_{28}O_6$	424.49	0	6	2	6	3	0.32	0.45
23	$C_{18}H_{14}O_5$	310.3	0	5	2	0	4	0.17	0.61
24	$C_{23}H_{22}O_5$	378.42	1	5	2	3	4	0.26	0.50
25	$C_{29}H_{32}O_6$	476.56	0	6	2	5	4	0.34	0.40
26	$C_{27}H_{28}O_5$	432.51	1	5	2	4	4	0.3	0.44
27	$C_{23}H_{22}O_6$	394.42	1	6	2	3	4	0.26	0.48
28	$C_{22}H_{20}O_6$	380.39	1	6	3	2	4	0.23	0.50
29	$C_{18}H_{16}O_7$	344.32	2	7	4	0	4	0.28	0.56
30	$C_{24}H_{24}O_6$	408.44	1	6	2	4	4	0.29	0.47
31	$C_{18}H_{16}O_7$	344.32	2	7	4	0	4	0.28	0.56
32	$C_{23}H_{24}O_7$	412.43	3	7	4	3	4	0.35	0.47
33	$C_{24}H_{26}O_6$	410.46	1	6	2	3	4	0.38	0.47
34	$C_{18}H_{14}O_5$	310.3	0	5	2	0	4	0.17	0.61

**35**	**$C_{23}H_{22}O_5$**	**378.42**	**1**	**5**	**2**	**3**	**4**	**0.26**	**0.50**
**36**	**$C_{29}H_{34}O_6$**	**478.58**	**0**	**6**	**3**	**8**	**3**	**0.34**	**0.40**
**37**	**$C_{23}H_{22}O_6$**	**394.42**	**0**	**6**	**3**	**2**	**4**	**0.26**	**0.48**
**38**	**$C_{19}H_{18}O_6$**	**342.34**	**1**	**6**	**2**	**1**	**4**	**0.32**	**0.56**
**39**	**$C_{29}H_{34}O_7$**	**494.58**	**0**	**7**	**4**	**9**	**4**	**0.34**	**0.39**
**40**	**$C_{48}H_{50}O_{13}$**	**834.9**	**2**	**13**	**6**	**10**	**7**	**0.33**	**0.46**
**41**	**$C_{47}H_{48}O_{13}$**	**820.88**	**2**	**13**	**7**	**9**	**7**	**0.32**	**0.47**
**42**	**$C_{26}H_{24}O_8$**	**464.46**	**2**	**8**	**1**	**6**	**5**	**0.27**	**0.59**
**43**	**$C_{24}H_{20}O_8$**	**436.41**	**2**	**8**	**2**	**4**	**5**	**0.21**	**0.63**
**44**	**$C_{24}H_{20}O_8$**	**436.41**	**2**	**8**	**2**	**4**	**5**	**0.21**	**0.63**

Table S2. Biophysiochemical properties of anti-inflammatory xanthones.

No	MR	TPSA	iLOGP	XLOGP3	WLOGP	MLOGP	Silicos-IT Log P	Consensus Log P	Ali Log S	ESOL Log S	Silicos-IT LogSw
1	72.55	100.13	2.36	2.42	2.07	0.02	2	1.77	-4.17	-3.52	-3.69
2	77.02	89.13	2.9	2.75	2.37	0.28	2.52	2.16	-4.28	-3.72	-4.39
3	75	68.9	2.58	2.55	2.67	0.82	3	2.32	-3.64	-3.52	-4.97
4	68.08	111.13	1.43	1.54	1.77	-0.24	1.49	1.2	-3.48	-2.97	-2.99
5	77.02	89.13	2.54	2.75	2.37	0.28	2.52	2.09	-4.28	-3.72	-4.39
6	70.53	79.9	2.36	2.77	2.37	0.57	2.48	2.11	-4.1	-3.67	-4.27
7	66.06	90.9	1.65	2.45	2.06	0.3	1.98	1.69	-4	-3.47	-3.57
8	133.17	50.44	5.16	8.94	7.18	4.62	8.22	6.82	-9.89	-7.99	-8.43
9	109.45	50.44	4.42	7.01	5.67	3.67	6.37	5.43	-7.88	-6.55	-7.21
10	119.99	100.13	4.14	6.27	5.09	2.19	5.52	4.64	-8.16	-6.35	-6.14
11	106.07	207.35	0.79	-0.21	-0.46	-2.58	-0.58	-0.61	-3.69	-2.63	-1.19
12	115.41	111.13	3.5	5.97	4.74	1.98	4.96	4.23	-8.08	-6.15	-5.45
13	100.74	89.13	3.55	4.67	3.88	1.39	4.25	3.55	-6.27	-5.13	-5.62
14	118.37	89.13	4.18	5.62	4.95	2.19	5.17	4.42	-7.25	-6.06	-6.31
15	137.06	90.9	4.78	8.16	6.81	3.48	7.16	6.08	-9.93	-7.61	-7.26
16	137.06	90.9	4.79	8.16	6.81	3.48	7.16	6.08	-9.93	-7.61	-7.26
17	141.53	79.9	5.01	8.49	7.11	3.67	7.73	6.4	-10.04	-7.83	-7.95
18	137.06	90.9	5.02	8.16	6.81	3.48	7.16	6.12	-9.93	-7.61	-7.26
19	91.8	111.13	2.08	4.02	3.28	0.93	3.17	2.69	-6.06	-4.71	-4.23
20	91.8	111.13	2.41	4.02	3.28	0.93	3.17	2.76	-6.06	-4.71	-4.23
21	119.99	100.13	4.21	6.27	5.09	2.19	5.52	4.66	-8.16	-6.35	-6.14
22	124.46	89.13	4.38	6.6	5.39	2.4	6.08	4.97	-8.27	-6.57	-6.83
23	88.16	79.9	2.93	3.73	3.43	1.48	3.36	2.99	-5.1	-4.56	-4.99
24	111.72	79.9	3.81	5.68	5.16	2.52	4.97	4.43	-7.12	-5.94	-6.23
25	142.09	89.13	5	7.55	6.46	3.13	7.01	5.83	-9.26	-7.52	-7.52
26	130.63	79.9	4.28	7.25	6.28	3.28	6.38	5.49	-8.75	-7.15	-7.05
27	113.52	89.13	3.91	5.44	4.56	1.98	4.66	4.11	-7.07	-5.87	-5.72
28	108.94	100.13	3.04	5.13	4.21	1.76	4.11	3.65	-6.98	-5.67	-5.03
29	90.16	120.36	2.49	1.7	1.6	-0.06	1.97	1.54	-3.84	-3.46	-3.82
30	117.54	89.13	4.2	6.01	4.75	2.19	5.46	4.52	-7.66	-6.24	-6.34
31	90.16	120.36	2.2	1.7	1.6	-0.06	1.97	1.48	-3.84	-3.46	-3.82
32	113.72	120.36	3.07	3.65	3.32	0.98	3.59	2.92	-5.87	-4.84	-5.06
33	117.9	89.13	4.09	6.01	4.93	2.26	5.45	4.55	-7.66	-6.32	-6.66
34	88.16	79.9	2.96	3.73	3.43	1.48	3.36	2.99	-5.1	-4.56	-4.99

**35**	**111.72**	**79.9**	**3.89**	**5.68**	**5.16**	**2.52**	**4.97**	**4.44**	**-7.12**	**-5.94**	**-6.23**
**36**	**143.55**	**100.13**	**4.87**	**8.13**	**6.82**	**3.13**	**7.27**	**6.04**	**-10.09**	**-7.7**	**-7.36**
**37**	**113.9**	**100.13**	**3.93**	**5.3**	**4.65**	**1.98**	**4.62**	**4.1**	**-7.15**	**-5.85**	**-5.62**
**38**	**94.18**	**89.13**	**3.03**	**4.09**	**3.42**	**1.24**	**3.67**	**3.09**	**-5.67**	**-4.89**	**-5.44**
**39**	**144.71**	**120.36**	**4.6**	**6.88**	**5.79**	**2.33**	**6.67**	**5.25**	**-9.22**	**-6.93**	**-6.79**
**40**	**237.25**	**209.49**	**6.79**	**10.51**	**8.94**	**2.05**	**9.92**	**7.64**	**-14.86**	**-11.32**	**-11.3**
**41**	**232.78**	**220.49**	**6.5**	**10.18**	**8.64**	**1.89**	**9.35**	**7.31**	**-14.75**	**-11.09**	**-10.62**
**42**	**125.86**	**96.59**	**3.99**	**4.08**	**4.26**	**1.21**	**4.7**	**3.65**	**-5.81**	**-5.33**	**-7.85**
**43**	**116.32**	**107.59**	**3.35**	**3.18**	**3.22**	**0.8**	**3.75**	**2.86**	**-5.11**	**-4.75**	**-6.77**
**44**	**116.32**	**107.59**	**3.45**	**3.18**	**3.22**	**0.8**	**3.75**	**2.88**	**-5.11**	**-4.75**	**-6.77**

Table S3. Violations of drug-likeness rules by the anti-inflammatory xanthones. For each compound, the type of violations of each rule was described.

No	Lipinski	Ghose	Veber	Egan	Muegge
**1**	0	0	0	0	0
**2**	0	0	0	0	0
**3**	0	0	0	0	0
**4**	0	0	0	0	0
**5**	0	0	0	0	0
**6**	0	0	0	0	0
**7**	0	0	0	0	0
**8**	1	2	0	1	1
**9**	0	1	0	0	1
**10**	0	0	0	0	1
**11**	2	1	1	1	3
**12**	0	0	0	0	1
**13**	0	0	0	0	0
**14**	0	0	0	0	1
**15**	0	2	0	1	1
**16**	0	2	0	1	1
**17**	0	2	0	1	1
**18**	0	2	0	1	1
**19**	0	0	0	0	0
**20**	0	0	0	0	0
**21**	0	0	0	0	1
**22**	0	0	0	0	1
**23**	0	0	0	0	0
**24**	0	0	0	0	1
**25**	0	2	0	1	1
**26**	0	2	0	1	1
**27**	0	0	0	0	1
**28**	0	0	0	0	1
**29**	0	0	0	0	0
**30**	0	0	0	0	1
**31**	0	0	0	0	0
**32**	0	0	0	0	0
**33**	0	0	0	0	1
**34**	0	0	0	0	0
**35**	0	0	0	0	1

**36**	0	2	0	1	1
**37**	0	0	0	0	1
**38**	0	0	0	0	0
**39**	0	3	0	0	1
**40**	3	4	1	2	5
**41**	3	4	1	2	5
**42**	0	0	0	0	0
**43**	0	0	0	0	0
**44**	0	0	0	0	0

MW – molecular weight, MR - molar refractivity, TNA - total number of atoms, PSA – polar surface area, HBA – hydrogen bond acceptor, HBD – hydrogen bond donor, RB - rotatable bonds, LOG P – considered log P value calculated by XLOGP3.

Table S4. Absorption and Metabolism parameters of anti-inflammatory xanthones.

No	GI absorption	BBB permeant	P-gp substrate	CYP1A2 inhibitor	CYP2C19 inhibitor	CYP2C9 inhibitor	CYP2D6 inhibitor	CYP3A4 inhibitor
1	High	No	No	Yes	No	No	Yes	Yes
2	High	No	No	Yes	No	Yes	Yes	Yes
3	High	Yes	No	Yes	No	Yes	Yes	Yes
4	High	No	No	Yes	No	No	Yes	Yes
5	High	No	No	Yes	No	Yes	Yes	Yes
6	High	No	No	Yes	No	No	Yes	Yes
7	High	No	No	Yes	No	No	Yes	Yes
8	Low	No	Yes	No	No	No	No	No
9	High	Yes	No	No	Yes	Yes	No	No
10	High	No	No	No	No	Yes	No	No
11	Low	No	No	No	No	No	No	No
12	High	No	No	Yes	No	Yes	No	No
13	High	No	No	Yes	No	Yes	No	No
14	High	No	No	No	Yes	Yes	No	No
15	Low	No	No	No	No	No	No	No
16	Low	No	No	No	No	No	No	No
17	Low	No	Yes	No	No	No	No	No
18	Low	No	No	No	No	No	No	No
19	High	No	No	Yes	No	Yes	Yes	No
20	High	No	No	Yes	No	Yes	Yes	No
21	High	No	No	No	No	Yes	No	No
22	High	No	No	No	No	Yes	No	No
23	High	No	No	Yes	No	Yes	Yes	Yes
24	High	No	No	Yes	Yes	Yes	No	Yes
25	Low	No	No	No	Yes	No	No	No
26	High	No	No	No	Yes	Yes	No	Yes
27	High	No	No	Yes	Yes	Yes	No	Yes
28	High	No	No	Yes	No	Yes	No	Yes
29	High	No	Yes	No	No	No	No	No
30	High	No	No	No	Yes	Yes	No	Yes
31	High	No	Yes	No	No	No	No	No
32	High	No	Yes	No	No	Yes	No	Yes
33	High	No	No	No	Yes	Yes	No	No
34	High	No	No	Yes	No	Yes	Yes	Yes

**35**	High	No	No	Yes	Yes	Yes	No	Yes
**36**	Low	No	No	No	No	No	No	No
**37**	High	No	No	No	No	Yes	No	No
**38**	High	No	No	Yes	Yes	Yes	Yes	Yes
**39**	Low	No	No	No	Yes	No	No	No
**40**	Low	No	Yes	No	No	No	No	No
**41**	Low	No	Yes	No	No	No	No	No
**42**	High	No	Yes	No	No	Yes	No	Yes
**43**	High	No	Yes	No	No	Yes	No	Yes
**44**	High	No	Yes	No	No	Yes	No	Yes
